# A Comprehensive Review of Thermosensitive Hydrogels: Mechanism, Optimization Strategies, and Applications

**DOI:** 10.3390/gels11070544

**Published:** 2025-07-14

**Authors:** Tianyang Lv, Yuzhu Chen, Ning Li, Xiaoyu Liao, Yumin Heng, Yayuan Guo, Kaijin Hu

**Affiliations:** 1 Xian Key Laboratory for the Prevention and Control of Stomatognathic System Disorders, School of Stomatology, Xi’an Medical University, Xi’an 710021, China; lvty@stu.jzmu.edu.cn (T.L.); chenyz@stu.jzmu.edu.cn (Y.C.); lin@stu.jzmu.edu.cn (N.L.); liaoxiaoyu@xiyi.edu.cn (X.L.); hengym@stu.jzmu.edu.cn (Y.H.); 2 Research Center of Dental and Maxillofacial Tissue Regeneration and Repair Technology, School of Stomatology, Xi’an Medical University, Xi’an 710021, China; 3 Engineering Research Center of Oral and Maxillary System Disease, School of Stomatology, Xi’an Medical University, Xi’an 710021, China

**Keywords:** thermosensitive hydrogels, mechanism, optimization strategy, delivery system, tissue engineering

## Abstract

Thermosensitive hydrogels undergo reversible sol-gel phase transitions in response to changes in temperature. Owing to their excellent biocompatibility, mild reaction conditions, and controllable gelation properties, these hydrogels represent a promising class of biomaterials suitable for minimally invasive treatment systems in diverse biomedical applications. This review systematically summarizes the gelation mechanisms of thermosensitive hydrogels and optimization strategies to enhance their performance for broader application requirements. In particular, we highlight recent advances in injectable thermosensitive hydrogels as a carrier within stem cells, bioactive substances, and drug delivery for treating various tissue defects and diseases involving bone, cartilage, and other tissues. Furthermore, we propose challenges and directions for the future development of thermosensitive hydrogels. These insights provide new ideas for researchers to explore novel thermosensitive hydrogels for tissue repair and disease treatment.

## 1. Introduction

Diseases, trauma, inflammation, and other factors can lead to damage in various tissues of the human body, strongly impacting patients’ daily lives and mental health. Currently, transplantation and pharmacological treatments are commonly used in clinical settings to address tissue damage [1,2]. However, transplantation faces challenges such as a high risk of infection and immunogenicity [3]. Traditional drug delivery methods, including oral and intravenous administration, often suffer from limitations such as short drug half-life, insufficient drug accumulation at target sites, and significant systemic side effects [4]. With continuous advancements in biomedical engineering, innovative delivery platforms—such as hydrogels [5,6], microneedles [7,8], and liposomes [9]—have been developed to achieve spatiotemporally controlled substance release, improve therapeutic precision, and promote functional tissue repair.

Among these platforms, hydrogels are widely used and highly regarded due to their structural similarity to natural tissues, excellent biocompatibility, and biodegradability [10]. However, traditional hydrogels often form irreversible crosslinked polymer networks [11], making in situ injection into small or delicate sites challenging. The dynamic nature of damaged tissues further limits the utility of these materials as they often fail to adapt rapidly to physiological changes [11], leading to suboptimal patient outcomes. Recent advances in stimuli-responsive hydrogels address these limitations by enabling precise reactions to environmental factors—including pH fluctuations, light exposure, temperature variations, mechanical stress, and electromagnetic fields—demonstrating significant potential for tissue regeneration [12].

Thermosensitive polymers, in particular, respond rapidly to localized temperature changes. Thermosensitive hydrogels, a subclass of smart hydrogels, have gained widespread attention due to their sensitivity to temperature and tunable phase transition behavior. These hydrogels permit in situ gelation, thereby enhancing site-specific delivery. The sol–gel transition triggered by external or internal temperature changes facilitates minimally invasive and targeted therapies [13]. Furthermore, since temperature is a benign stimulus, gelation can occur without the use of harmful solvents, which simplifies the preparation process [14]. Importantly, the thermosensitive hydrogel gradually degrades in vivo, eliminating the need for surgical removal and improving patient convenience.

Thermosensitive hydrogels offer distinct advantages as carriers for localized therapeutic delivery [15,16], making them a prominent focus in tissue engineering research. Despite their widespread exploration, systematic reviews on their role in repairing tissue defects remain scarce. This review examines the gelation mechanisms underlying thermosensitive hydrogels and evaluates current optimization approaches. It further analyzes recent advances in delivering stem cells, drugs, and bioactive molecules via injectable thermosensitive hydrogels across bone, cartilage, and other tissues, providing critical perspectives for future hydrogel design.

## 2. Phase Transition and Gelation Mechanisms of Thermosensitive Hydrogel

**Figure 1:**
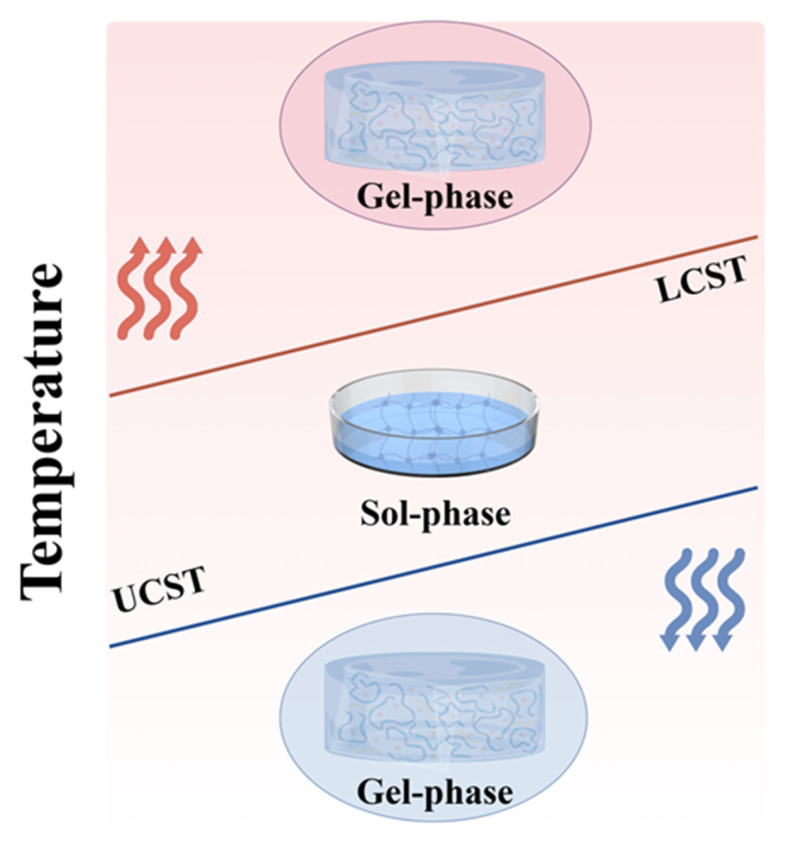
Diagram of the LCST and UCST phase transitions of thermosensitive hydrogels (The arrows indicate heating and cooling.).

The advancement of biomaterials science and tissue engineering has stimulated extensive research into thermoresponsive hydrogels. These smart polymer networks exhibit volume phase transition behavior upon thermal stimulation, with the corresponding temperature referred to as the volume phase transition temperature [17]. Based on their phase transition temperature, thermosensitive hydrogels can be classified into two categories: those with a lower critical solution temperature (LCST) and those with an upper critical solution temperature (UCST) (Figure 1). The LCST marks the temperature at which a material transitions from one phase to another upon heating. Heating above this threshold induces molecular precipitation and a subsequent sol–gel transition [18,19]. Conversely, the UCST follows an inverse pattern, with polymers gelling at lower temperatures and dissolving when heated beyond this point [20]. Although UCST-type thermoresponsive polymers, such as agarose and cold glue, exist, their limited responsiveness at physiological temperatures restricts their biomedical utility [21]. By contrast, LCST-type polymers show greater promise for biomedical applications and have consequently attracted more research attention [22].

Thermodynamically speaking, the contraction of polymers to form gels is attributed to the interplay between enthalpy, entropy, and temperature. At high temperatures, the increase in entropy of water molecules dominates, leading to a decrease in free energy (ΔG = ΔH − ΔS < 0) and resulting in phase separation. Conversely, at low temperatures, the exothermic effect (ΔH < 0) compensates for the cost of entropy reduction (ΔG = ΔH − TΔS < 0), leading to phase separation [23]. Specifically, LCST-type hydrogels are predominantly driven by entropy, while UCST-type hydrogels are primarily driven by enthalpy. This difference arises from the temperature dependence of polymer-solvent interactions and the competing roles of enthalpy and entropy in determining the free energy [24].

**Figure 2:**
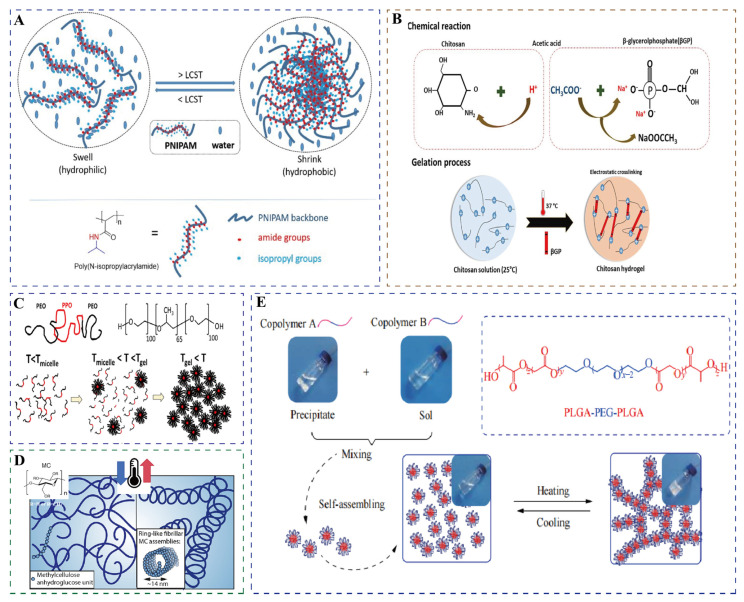
Schematic diagram of gelation of common thermosensitive hydrogels. (**A**) PNIPAm. Used with permission of the Royal Society of Chemistry, from Thermoresponsive polymers and their biomedical application in tissue engineering—a review, Falko Doberenz(s), 4, 8 and 2020 of copyright [26]. (**B**) CS/β-GP. Reprinted from *International Journal of Biological Macromolecules*, 164, Nourollah Rezaei(s), Antimicrobial peptides-loaded smart chitosan hydrogel: Release behavior and antibacterial potential against antibiotic resistant clinical isolates, 855–862, copyright (2020), with permission from Elsevier [33]. (**C**) F127 (T represents temperature). Reprinted with permission from [35]. Copyright 2021, American Chemical Society. (**D**) Methylcellulose (The arrows indicate heating and cooling.). Adapted with permission from [37]. Copyright 2018, American Chemical Society. (**E**) PLGA-PEG-PLGA. Reprinted with permission from [38].

Thermosensitive hydrogels exhibit remarkable molecular diversity in their structural features and gelation mechanisms. Among them, N-isopropylacrylamide (NIPAm) stands out as the most representative thermosensitive monomer. Its polymer, poly(N-isopropylacrylamide) (PNIPAm), possesses a distinctive amphiphilic molecular architecture comprising hydrophilic amide groups and hydrophobic isopropyl side chains [25]. The temperature-responsive behavior originates from dynamic interactions between functional groups and solvent molecules. At temperatures below the LCST, hydrogen bonding between amide groups and water molecules maintains chain expansion, while heating above the LCST triggers chain collapse and subsequent hydrophobic aggregation through hydrogen bond dissociation and hydrophobic interactions (Figure 2A) [26,27]. In addition to NIPAm, poly(2-dimethylaminoethyl methacrylate) serves as a typical thermosensitive monomer. The tertiary amine groups in its side chains undergo dynamic hydrophilic–hydrophobic transitions in response to temperature variations, thereby inducing phase separation [28]. Similarly, 2-(2-methoxyethoxy)ethyl methacrylate (MEO_2_MA) and oligo(ethylene glycol) methacrylate (OEGMA) are common thermosensitive monomers. The thermoresponsive behavior of MEO_2_MA originates from the dissociation and reconstruction of dynamic hydrogen-bonding networks between ether oxygen atoms in its side chains and water molecules upon temperature changes [29], whereas OEGMA achieves thermosensitivity through hydrogen bond interactions among ether oxygen atoms, methylene groups in the polymer chains, and water molecules [30]. Furthermore, when the OEGMA monomer undergoes polymerization, it can undergo copolymerization with other monomers, thereby acquiring the ability to control the LCST [31].

Other important thermosensitive systems include chitosan (CS), which requires polyol salts, such as β-sodium glycerophosphate (β-GP), to achieve thermosensitivity [32]. Under physiological pH conditions (pH = 7–7.4), polyol molecules form a protective hydrophobic layer through hydrogen bonding at low temperatures, maintaining the solution state. When the temperature increases to 37 °C, thermodynamic forces disrupt this hydrophobic layer, leading to a sol–gel transition via enhanced hydrophobic interactions (Figure 2B) [33,34]. Pluronic F127 (F127) copolymers, with their poly(ethylene oxide)-b-poly(propylene oxide)-b-poly(ethylene oxide) (PEO-PPO-PEO) sequence, undergo dehydration-driven micellization of the hydrophobic PPO blocks above the critical micellar temperature. Further heating beyond the critical gelation temperature induces intermicellar entanglement, leading to a sharp increase in viscosity and the formation of a gel network (Figure 2C) [35]. Notably, F127 self-assembles into a face-centered cubic ordered structure at 37 °C [36]. Cellulose derivatives, such as methylcellulose (MC), derive their thermosensitivity from β-1,4-glycosidic bonded glucose backbones [22]. Heating disrupts hydrogen bonds, allowing methoxy groups to crosslink through strong hydrophobic interactions, which promotes the self-assembly of random-coiled molecular chains into persistent fibrils approximately 14 nm in diameter, ultimately forming a stable three-dimensional hydrogel network (Figure 2D) [37]. The poly (D, L-lactide-co-glycolide)-b-poly (ethyleneglycol)-b-poly (D, L-lactide-co-glycolide) triblock copolymer system, abbreviated as PLGA-PEG-PLGA, exhibits temperature responsiveness through dehydration of the PEG segment upon heating, which enhances hydrophobicity and promotes micelle aggregation and three-dimensional network formation(Figure 2E) [38,39].

The common feature of these systems lies in the thermally induced phase transition driven by physical or chemical interactions such as dynamic reorganization of hydrogen-bond networks, hydrophobic interactions, and solute solvent interactions. Currently, some characterization techniques, such as dynamic light scattering, scanning electron microscopy, Fourier transform infrared spectroscopy, and Raman spectroscopy, provide multi-scale insights into the gel mechanisms and structure performance relationships of these complex systems, laying the foundation for the development of new thermosensitive hydrogels and the exploration of the gelation mechanisms [40,41].

## 3. Optimization Strategies of Thermosensitive Hydrogels

**Figure 3:**
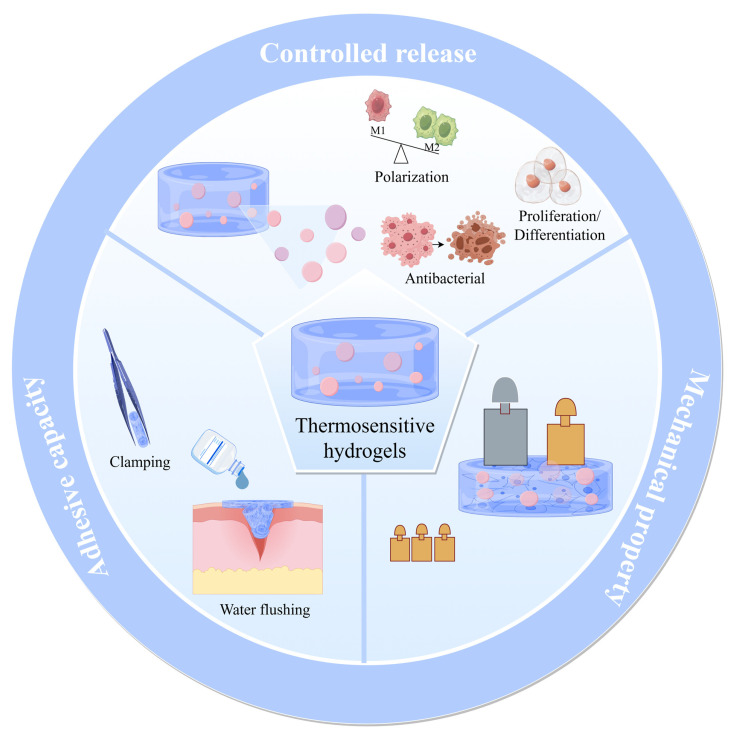
The optimized superior performance of thermosensitive hydrogels.

Thermosensitive hydrogels respond intrinsically to temperature changes, yet both natural and synthetic variants suffer from inherent drawbacks, including poor stability, inadequate mechanical properties, and uncontrolled release profiles. To address these challenges, optimization strategies—including modifications in crosslinking methods, internal encapsulation, three-dimensional printing, and multi-stimuli responsiveness—have been employed to enhance the performance of thermosensitive hydrogels and expand their applications in tissue engineering (Figure 3).

### 3.1. Crosslinking Method

Physical crosslinking is a fundamental approach in the preparation of hydrogels. Physical crosslinking involves the formation of three-dimensional networks through reversible, non-covalent interactions between polymer chains, including but not limited to hydrophobic associations, electrostatic forces, hydrogen bonding, van der Waals interactions, and physical chain entanglements during gelation [42,43]. Thermosensitive hydrogels formed through physical crosslinking can undergo temperature-induced phase transformation without the need for coupling agents or organic solvents, offering excellent biological safety. However, the thermosensitive hydrogels prepared solely through physical crosslinking often exhibit limited functional properties [44] (including low mechanical strength, weak adhesion, burst release of encapsulated substances, slow gelation kinetics, etc.) and fail to achieve the material diversity required for broader biomedical applications. Therefore, selecting an appropriate crosslinking method is a crucial strategy to further enhance the performance and functionality of thermosensitive hydrogels.

Chemical crosslinking relies on crosslinking agents to mediate irreversible covalent bonding (e.g., through addition or condensation reactions), resulting in a permanent hydrogel network with chemically bonded polymer chains [45,46]. Compared with thermosensitive hydrogels formed through physical crosslinking, those synthesized via chemical crosslinking exhibit substantially greater mechanical robustness and enhanced structural stability. Furthermore, reactions between chemical bonds and chemical groups can further improve the material’s adhesive properties [47]. However, residual crosslinking agents such as formaldehyde and glutaraldehyde may exert adverse effects on biological tissues. In contrast, plant-derived extracts—including genipin [48], proanthocyanidins [49], and tea polyphenols [50]—serve as natural crosslinkers that are devoid of toxicity and side effects, rendering them highly favored by researchers. Representative examples of widely utilized chemically crosslinked thermosensitive hydrogels include polyether F127 diacrylate (F127DA) [51] and polyethylene glycol diacrylate [52], which have become benchmark materials in this field due to their tunable network properties.

Metal coordination crosslinking is a novel strategy for constructing thermosensitive hydrogels, relying on the dynamic interactions between metal ions and polymer coordination groups. By modulating the strength and density of coordination bonds in response to temperature changes, these hydrogels achieve reversible contraction or swelling of the gel network [53,54]. The core mechanism involves the formation of dynamic coordination bonds between metal ions (such as Cu^2+^, Zn^2+^, and Al^3+^) and coordination groups like carboxylic acid, catechol, and amino groups on the polymer chains [53,55,56]. As the temperature increases, the bond energy of these coordination interactions weakens or dissociates, reducing the number of crosslinking points, relaxing the gel network, and promoting water absorption and swelling. Conversely, the coordination bonds re-form when the temperature decreases, leading the network to contract and expel water. For instance, the coordination system between Fe^3+^ and catechol groups (mimicking the structure of mussel adhesive proteins) dissociates above body temperature, imparting the material with significant thermosensitive volume phase transition characteristics [57,58]. Additionally, the strength of the coordination bonds can be precisely controlled by adjusting the metal valence state, ligand type, and environmental pH, allowing for the customization of the gel’s response temperature and mechanical properties. Some metal ions can pose a threat to tissue safety, so reducing their toxicity through controlled concentrations or biological methods, such as transformation and adsorption, remains essential. Beyond tissue engineering, temperature-sensitive hydrogels with metal coordination crosslinking also function effectively in wearable devices [59,60].

Photothermal synergistic crosslinking is achieved by copolymerizing monomers containing thermosensitive groups with those containing photosensitive groups or by introducing photosensitive groups into the terminal groups of polymers through chemical methods. Local polymerization is then initiated via ultraviolet (UV) illumination, forming a temperature-responsive network [61,62]. In recent years, upconversion nanoparticles have emerged as an effective means of converting external near-infrared light into internal light, demonstrating the potential of near-infrared light to replace UV in triggering drug release [63,64]. This innovation provides new insights for the development of photothermal synergistic hydrogels. Numerous experiments have introduced polydopamine nanoparticles, gold nanorods, or nano-liposomes into thermally responsive hydrogels. Upon exposure to near-infrared light, these hydrogels alter their local crosslink density, thereby enhancing spatiotemporal precision and synergistic thermotherapeutic effects and enabling the controlled release of internal gel contents [65,66]. The above-mentioned crosslinking methods are summarized in Table 1.

### 3.2. Internal Encapsulation

Although thermosensitive hydrogels are highly diverse, conventional thermosensitive hydrogels cannot promote tissue healing. Considering that these hydrogels consist of three-dimensional polymer networks with high water content, they provide a moist microenvironment that enhances biocompatibility. Furthermore, their gradual degradation after implantation and these characteristics make them particularly suitable as carrier materials.

To fulfill the three essential components of tissue engineering, researchers typically incorporate seed cells and growth factors (GFs) into thermosensitive hydrogels [67]. Seed cells may include autologous, allogeneic, stem, or genetically modified cells [68]. The thermosensitive hydrogel provides a microenvironment similar to the natural extracellular matrix (ECM), which facilitates cell encapsulation and survival, enabling the delivery of exogenous cells to the injury site and promoting tissue repair. GFs are bioactive molecules that regulate cellular behaviors by binding to cell surface receptors, activating signaling pathways, and modulating cell proliferation and differentiation [69]. As the thermosensitive hydrogel degrades, the encapsulated GFs are gradually released, promoting tissue healing and regeneration. Consequently, cells and GFs represent the most common internal cargoes within thermosensitive hydrogels.

Drugs with antibacterial or anti-inflammatory effects are also frequently loaded into thermosensitive hydrogels. This is attributed to tissue damage, where bacteria and inflammation often play a role, hindering the subsequent tissue repair and regeneration. By loading drugs onto hydrogels and achieving sustained release at the defect site, the effects of antibacterial, anti-inflammatory, and pro-regenerative impact, such as promoting cell proliferation and differentiation, are particularly significant. In this regard, thermosensitive hydrogels offer distinct advantages. Because they contain hydrophilic polymers and adapt well to the shape of the loaded drug, drug incorporation is relatively easy. Water-soluble drugs can be mixed with high-molecular-weight aqueous solutions to prepare hydrophilic drug-loaded gels, which enable the diffusion of drugs from the matrix and achieve in situ release functionality [70]. However, for some hydrophobic drugs, the direct use of gel encapsulation results in a poor utilization rate. By combining drugs with nanomaterials, a stable suspension can be formed in thermosensitive hydrogels, enabling the controlled release of small doses and addressing the issue of poor water solubility [71]. Notably, liposomes exhibit low cytotoxicity and immunogenicity, serving as an effective delivery platform for both hydrophobic and hydrophilic molecules while protecting their biological activity from degradation [72]. Combining drug-loaded nanomaterials or liposomes with thermosensitive hydrogels facilitates controlled drug release, presenting a potential strategy for targeted delivery to inflammatory tissue injury sites.

Moreover, studies have demonstrated that incorporating materials such as hydroxyapatite, calcium carbonate, and decellularized bone matrix into thermosensitive hydrogels can significantly enhance their mechanical properties. The calcium, phosphorus, and other bioactive components released from these materials can meet the functional demands of the deficient areas, promote the healing of bone tissues, and enhance bone strength [73,74].

### 3.3. Three-Dimensional Printing Technique

Three-dimensional (3D) printing, also known as additive manufacturing, is a layer-by-layer fabrication technology widely applied across multiple disciplines. 3D bioprinting, an advanced technique that involves the spatially controlled deposition of living cells, biomaterials, pharmaceuticals, GFs, and genes layer by layer, has rapidly evolved in recent years and demonstrated extensive utility in various biomedical applications [75]. However, identifying suitable bioinks remains a significant challenge in 3D bioprinting, reflecting the delicate balance between reproducible additive manufacturing and biological requirements [76].

Thermosensitive hydrogels exhibit intrinsic properties that render them promising candidates for bioinks. However, their precursor solutions often exhibit poor rheological characteristics, including low viscosity and shear resistance, which compromise the shape fidelity of printed constructs. This limitation can be mitigated by incorporating nanocellulose or nanoclay into the hydrogel matrix. During printing, the sol state of the hydrogel is extruded and undergoes instant gelation upon exposure to physiological temperatures. This occurs because nanocellulose bridges micelles through intermolecular hydrogen bonds and hydrophobic interactions, enabling physical crosslinking [77]. This approach ensures high shape fidelity and enables controlled drug release, thereby facilitating personalized therapeutic applications. In addition, the challenge of low-viscosity hydrogels being difficult to shape can be addressed through the freeform reversible embedding of suspended hydrogels (FRESH) method. This approach utilizes gelatin particles to prepare a supporting bath, allowing the printed hydrogel structure to maintain its position without collapsing [78]. It is particularly suitable for constructing multifunctional hydrogels, such as human cardiac capillaries [79]. The prepared thermosensitive hydrogel is used as the bioink and printed via the FRESH method. Upon heating to 37 °C, the gelatin supporting bath melts, yielding the final material. This technique eliminates the need for secondary crosslinking, preserves bioactivity, simplifies the fabrication process, and lays the foundation for large-scale production and clinical translation [80].

Two-photon polymerization (2PP), one of the most precise 3D printing technologies, enables the fabrication of high-resolution materials in conjunction with computer-aided design tools. Compared with the conventional 3D printing UV polymerization method, 2PP does not require toxic reagents or UV exposure and can rapidly produce flexible, high-precision structures with complex geometries [81,82]. Thermosensitive hydrogel microrobots fabricated via 2PP exhibit superior swelling properties, facilitating efficient drug release [83]. In addition, grafting thermosensitive P (NIPAMx-co-NtBAMy) side chains enabled the formulation of a functional bioink. This composition forms a weak gel during printing, shielding cells from shear-induced damage. Subsequent hydrophobic interactions enhance mechanical stability, facilitating cell migration and spheroid formation while establishing an optimal growth microenvironment [84]. Overall, the thermosensitive hydrogel produced by 3D printing has excellent mechanical properties and the ability to control drug release.

### 3.4. Multiple Stimulus Response

The human body maintains a constant core temperature, yet microenvironmental variations at tissue injury sites remain clinically unpredictable. Thermosensitive hydrogels that integrate temperature responsiveness with additional stimulus-triggered mechanisms show enhanced drug release profiles and therapeutic efficacy. In cancer therapy, the characteristic acidic tumor microenvironment enables pH-responsive drug release through the protonation of chitosan’s amino groups [85]. Magnetic responsiveness can be achieved by incorporating Fe_3_O_4_ nanoparticles into PNIPAm-based hydrogels, thereby combining magnetic targeting with thermally controlled drug delivery for precision oncology applications [86]. For cutaneous wound repair, in situ-forming CS gels functionalized with gallic acid modulate anti-inflammatory and regenerative drug release through reactive oxygen species (ROS) scavenging mechanisms [87]. Such ROS-responsive thermosensitive hydrogels have also proven effective in other tissue regeneration contexts [88,89]. When combined with previously described photothermal crosslinking techniques, these systems successfully integrate multiple stimuli-responsive modalities—including light, pH, ROS, and magnetism—demonstrating both practical utility and translational potential across diverse clinical applications.

## 4. Application of Thermosensitive Hydrogel in Tissue Repair and Treatment

**Figure 4:**
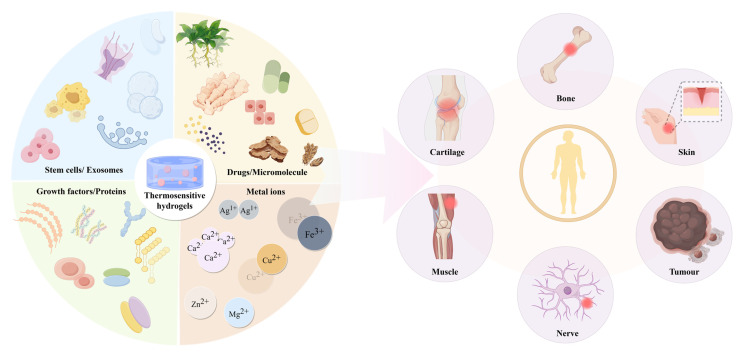
Thermosensitive hydrogel carriers and their application as delivery systems in tissue repair and treatment.

Tissue engineering integrates multidisciplinary approaches to create biological substitutes that safely and effectively repair or replace damaged human tissues. While some tissue-engineered products have achieved clinical success, persistent challenges include optimizing material biocompatibility, cellular stability, and functional tissue integration. Thermosensitive hydrogels provide a versatile platform for delivering stem cells, pharmaceuticals, and bioactive components, including exosomes, peptides, and metal ions. These hydrogels combine intelligent stimuli-responsiveness with biocompatibility, injectability, and controlled release properties, substantially improving therapeutic outcomes in both drug delivery and in situ tissue regeneration applications. Their unique characteristics make them particularly suitable for addressing diverse tissue defects (Figure 4).

### 4.1. Application of Thermosensitive Hydrogel in Bone Tissues

In the clinical treatment of bone defects, autologous, allogeneic, and xenogeneic bone transplantation are commonly used methods; however, their development is limited by issues such as insufficient donor sites and immunogenicity [2,90]. Due to its excellent properties, thermosensitive hydrogel-based delivery systems are widely used in bone tissue repair.

Stem cells, present in embryos and fetuses as undifferentiated cells, possess self-replication capabilities and are multipotent, meaning they can differentiate into various types of functional cells [91]. Thermosensitive hydrogels achieve tissue repair and regeneration by delivering stem cells. Guo et al. [92] prepared mesenchymal stem cell (MSC)–endothelial cell (EC) mixed spheroids using an AggreWell^™^400 24-well plate, then uniformly dispersed them in F127DA to create a composite bone repair material. The thermosensitive hydrogel increased in viscosity when injected into rat tooth extraction sockets, ensuring stable retention within the alveolar cavity. Subsequent photopolymerization enhanced material stability, while the encapsulated spheroids demonstrated active 3D cellular migration, exhibiting both pro-angiogenic and osteogenic potential during the early stages. With the advancement of the research field, extracellular vesicles (EVs) produced by stem cells, a bioactive substance, have emerged as a viable option for treating bone defects [93]. Ming et al. [94] investigated a hydrogel made of F127, CS, and crosslinked hyaluronic acid (c-HA) modified with calcium peroxide (CPO) and ascorbic acid (AsA) and loaded with bone marrow mesenchymal stem cell-derived extracellular vesicles (BMSCs-EVs). The crosslinking and modification processes yielded a gel with more uniform and compact pores, providing effective channels for sustained oxygen and EV release. This PCc/CPO/AsA/sEVs hydrogel material significantly promoted the repair and regeneration of alveolar bone defects. Wu et al. [95] isolated EVs from BMSCs and mixed them with CS/β-GP hydrogel. Their study demonstrated that hydrogel degradation was accompanied by sustained release of BMSC-derived EVs, wherein EV-encapsulated miR-21 promoted angiogenesis through targeting the SPRY2 gene. The osteogenic efficacy of this hydrogel was shown to be significant in critical-sized calvarial defects.

**Figure 5:**
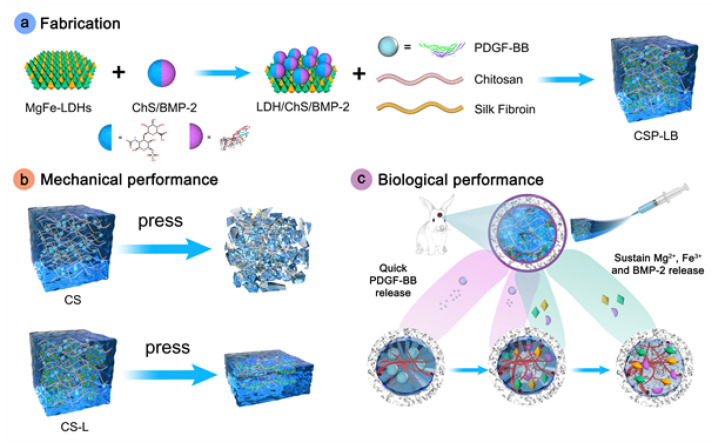
Preparation and functional schematic diagram of CSP-LB hydrogel. Reprinted with permission from [98].

During the delivery process, GFs are prone to being degraded into proteins by enzymes [96]. However, the hydrogel absorbs water and expands, forming a hydrophilic environment that prevents the inactivation of GFs [97]. This enables the precise release at the damaged site. Lv et al. [98] developed a dual-mode hydrogel designated as CSP-LB. This composite material consists of a CS/silk fibroin(SF) hydrogel loaded with platelet-derived growth factor-BB (PDGF-BB) as the matrix, incorporated with bone morphogenetic protein-2 (BMP-2)-functionalized two-dimensional layered double hydroxide (MgFe-LDH) nanosheets. The addition of nanosheets significantly improved the mechanical strength of the CSP-LB hydrogel, while simultaneously enhancing its thermosensitivity and reducing the gelation temperature. The weak electrostatic attraction between PDGF-BB and CS promoted rapid release, whereas the strong interaction between BMP-2 and LDH resulted in sustained release, maintaining prolonged growth factor activity during treatment. The hydrogel demonstrated superior osteogenic properties in critical-sized calvarial defects (Figure 5). Basic fibroblast growth factor (bFGF) induces BMSC differentiation into neuronal cells while enhancing vascular endothelial growth factor (VEGF) expression [99,100], although its therapeutic potential is constrained by rapid metabolic clearance and instability. Chu et al. [101] overcame these limitations by grafting NIPAm onto sodium alginate chains through PNIPAm-based free radical copolymerization, then integrating bFGF/dexamethasone (Dex)-loaded liposomes prepared via thin-lipid film hydration. The resulting thermosensitive bFGF/Dex lipo-gel combines manufacturing scalability with biocompatibility. Within this system, hydrophilic bFGF is encapsulated in the liposomal aqueous phase, while hydrophobic Dex is incorporated into the lipid bilayer, resulting in sequential release kinetics. This dual-compartment architecture sustained prolonged bFGF elution alongside controlled Dex delivery. In cranial defect models, the lipo-gel significantly enhanced angiogenesis, neural regeneration, and osseous repair.

**Figure 6:**
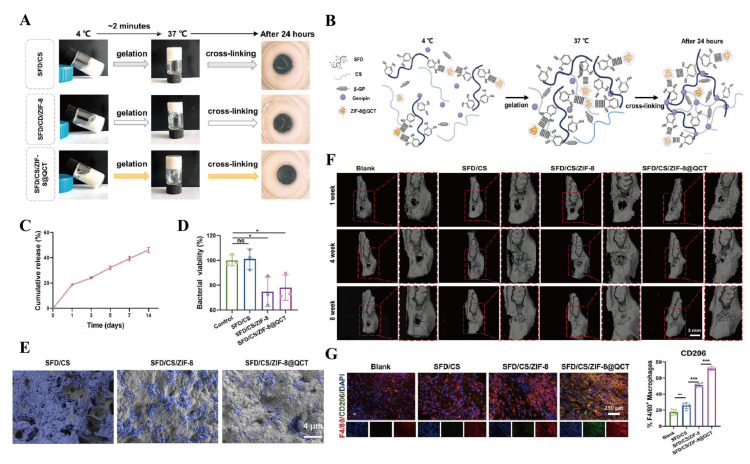
SFD/CS/ZIF-8@QCT hydrogel for bone tissue engineering. (**A**) Optical photograph of SFD/CS/ZIF-8@QCT after gelation for 24 h. (**B**) Molecular crosslinking diagram of SFD/CS/ZIF-8@QCT. (**C**) The sustained-release effect of QCT. (**D**) Quantitative analysis of the viability of *P. gingivalis* after 24 h of treatment. (**E**) SEM images of *P. gingivalis* after 48 h of treatment. (**F**) The 3D reconstructed images of inflammatory alveolar socket repair.(The red squares refers to the local magnification of alveolar bone defects implanted with different composite hydrogels.) (**G**) CD206 immunofluorescence staining and quantitative analysis. Data are presented as mean ± SD. * *p* < 0.05, ** *p* < 0.01, and *** *p* < 0.001; ns, not significant. Reproduced with permission from Ref. [104], Elsevier.

Bacteria and inflammatory microenvironments are common etiological factors of bone defects, making infection control particularly crucial. Thermosensitive hydrogels with strong adhesive properties play a pivotal role in the repair of inflammatory bone defects. These systems are typically administered via injection, filling, or surgical implantation to achieve localized drug release within the defect area, thereby exerting anti-inflammatory, antibacterial, and osteogenic effects. FDA-approved F127 thermosensitive hydrogel serves as an effective carrier for anti-inflammatory and antibacterial agents [102], although its limited bone adhesion restricts clinical utility. Almoshari et al. [103] addressed this limitation by developing a pyrophosphorylated Pluronic F127 composite incorporating BIO, a glycogen synthase kinase 3 beta inhibitor with dual anti-inflammatory and osteogenic activity, which demonstrated stable bone adhesion. The micellar encapsulation system enhanced the solubility of hydrophobic BIO, while enabling controlled drug release through a combination of surface erosion and diffusion mechanisms. In vivo studies confirmed that the PF-127-BIO hydrogel promoted the expression of β-catenin-positive cells, reduced inflammatory cell infiltration, and protected the alveolar bone and periodontal ligament structures. Similarly, to achieve the three interrelated objectives of antibacterial action, anti-inflammatory effects, and periodontal regeneration in periodontitis treatment strategies, Yang et al. [104] developed a thermosensitive and adhesive SFD/CS/ZIF-8@QCT hydrogel. This material was prepared by mixing dopamine-modified SF (SFD) with CS/β-GP and encapsulating quercetin (QCT)-modified zeolitic imidazolate framework-8 (ZIF-8). Studies demonstrated that after 24 h of gelation at physiological temperature, the free amino groups in CS and SFD underwent covalent double-crosslinking under the action of genipin, further enhancing the material’s adhesive properties. This modification provided optimal conditions for prolonged retention within periodontal pockets, resistance to gingival crevicular fluid, and sustained release of QCT and Zn^2+^ from ZIF-8. The SFD/CS/ZIF-8@QCT system meets the criteria for antibacterial, anti-inflammatory, and osteogenic activity. It exhibits excellent repair outcomes in inflammatory alveolar bone defects and holds great potential for clinical application (Figure 6).

Bone defects that occur in patients with diabetes due to the influence of a high-glucose environment are often difficult to repair. Currently, the conventional treatment of diabetic bone defects requires a multidisciplinary approach, including systemic and local infection control, blood glucose regulation, and promotion of local bone growth [105]. However, the delivery of bioactive substances through thermosensitive hydrogels provides a new strategy for treating diabetic bone defects. A bioactive compound called Coenzyme Q10 (CoQ10), used to improve alveolar sockets, can protect cells from damage and promote osteoblast proliferation and differentiation [106]. Ghanem’s team [107] developed a clinically viable CoQ10/collagen thermosensitive hydrogel by dispersing CoQ10 into an F127 solution and mixing it with a neutralized collagen solution (pH 7.4). The researchers recruited 18 patients with type II diabetes mellitus who were scheduled for single or multiple tooth extractions and implanted the composite material post-extraction. Histological and RT-qPCR analyses confirmed enhanced osteogenic activity in the CoQ10-treated group. The controlled CoQ10 release also markedly reduced post-operative pain and inflammation in these patients. The successful development and clinical implementation of this material broadens therapeutic approaches for diabetic bone defect repair. Surface area and diffusion rate are the main factors affecting the release of protein materials from hydrogels, and generally, the release rate is relatively fast. However, incorporating proteins into a crystalline matrix can further slow the release rate. Sheng et al. [108] adopted an innovative approach: they loaded bone morphogenetic protein-4 (BMP-4) onto graphene oxide (GO) and encapsulated it using the porcine small intestinal submucosa (SIS) as an ECM material, thus preparing a GB@SIS hydrogel with temperature-sensitive properties. The presence of GO and the external hydrogel introduces an additional step for BMP-4 release, thereby achieving its slow and uniform release. BMP-4 synergizes with ECM components in SIS to effectively suppress the NLRP3 signaling pathway and upregulate the expression levels of adhesion-related proteins. Through these mechanisms, the hydrogel demonstrates remarkable efficacy in modulating immune responses and promoting multifunctional osteogenesis, providing robust support for the repair of diabetic bone defects. The application scope and structural design of thermosensitive hydrogels in bone tissues are shown in Table 2.

### 4.2. Application of Thermosensitive Hydrogels in Cartilage Tissues

Trauma, functional degeneration and inflammation are all causes of cartilage damage. Among them, inflammatory factors are the most common. Osteoarthritis (OA) is characterized by subchondral bone remodelling, synovial inflammation, and cartilage degeneration, making it one of the most prevalent chronic degenerative joint diseases [119]. Thermosensitive hydrogels exhibit significant potential in the treatment of OA. When the thermosensitive hydrogel delivery system is locally injected into the joint cavity, it gels at physiological temperatures to achieve material retention, thereby prolonging the drug release time, enhancing drug targeting, and enabling the repair and regeneration of the joint cartilage area [120].

Radiosynoviorthesis refers to the intra-articular administration of radiopharmaceutical agents to alleviate synovial inflammation and reduce cartilage damage [121]. However, potential leakage of radioactive isotopes remains a challenge in current practice. Liu et al. [122] developed a thermosensitive hydrogel as a novel radiopharmaceutical delivery system (^177^Lu/AMP@CG). The system utilized self-assembled nanoparticles composed of adenosine monophosphate (AMP) ligands and the isotope-labeled metal ion ^177^Lu^3+^, which were incorporated into a CS/β-GP hydrogel to achieve prolonged intra-articular retention post-injection. The nanoparticles exhibited sustained-release characteristics, maintaining 86% of their radioactivity after 16 days in the joint space. This ^177^Lu/AMP@CG hydrogel reduced radioactive leakage risks while simultaneously delivering anti-inflammatory and chondroprotective effects, markedly enhancing OA treatment outcomes. N-acetyl-D-glucosamine (GlcNAc) is a nutritional supplement that can activate chondrocytes. Chang et al. [123] prepared a continuous delivery of GlcNAc based on F127, providing a promising strategy for enhancing cartilage repair. This hydrogel system prolonged GlcNAc release, improving its therapeutic effects in cartilage injury models. In rats with articular cartilage damage, intra-articular injection of GlcNAc hydrogel significantly improved joint function, as evidenced by increased step length, reduced foot angle, and delayed fall time. The hydrogel promoted cartilage regeneration by upregulating Sox9 and collagen II, while reducing chondrocyte apoptosis by approximately 50% through the suppression of cleaved caspase 3 and caspase 8. This hydrogel is easy to prepare, safe, and reliable and is expected to be further utilized in the clinical treatment of cartilage defects.

**Figure 7:**
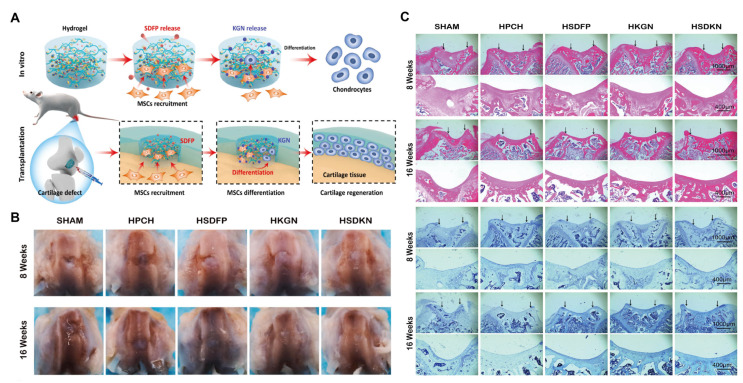
HSDKN hydrogel for cartilage tissue engineering. (**A**) Schematic diagram of HSDKN hydrogel promoting stem cell recruitment and cartilage differentiation through bioactive substances release. (**B**) Macroscopic diagram of knee cartilage healing. (**C**) Histological evaluation of knee cartilage healing-H&E staining and toluidine blue staining (Black arrows indicate the defect area.) (HSDFP, HPCH mix with 2 μg/mL SDFP; HKGN, HPCH/OCD-KGN 200; HSDKN, HPCH/OCD-KGN 200 mix with μg/mL SDFP;). Reproduced with permission from Ref. [124], Elsevier.

Thermosensitive hydrogels that achieve stem cell recruitment by releasing bioactive substances represent an innovative strategy for promoting tissue regeneration, especially in challenging environments such as articular cartilage defects. Yuan et al. [124] established a dual-drug delivery system, HSDKN, containing stromal cell-derived factor-1α-like polypeptide (SDFP) and kartogenin (KGN). This material first loads KGN into the hydrophobic cavity of aldehyde-modified β-cyclodextrin (OCD) through the interaction between the host and guest. Then, the two are fixed by the Schiff base reaction between the aldehyde in OCD and the amino groups in the hydroxypropyl chitosan hydrogel (HPCH). Subsequently, SDFP is physically mixed into the gel to prepare the final drug-loaded gel. The release of the loaded drugs synergistically promotes stem cell recruitment and cartilage differentiation. SDFP was critical in early-stage MSCs homing, addressing the limited endogenous cell recruitment in cartilage injuries. Meanwhile, KGN promoted chondrogenic differentiation, synergistically improving cartilage repair. In vivo studies confirmed the ability to accelerate cartilage regeneration in rat models (Figure 7). This dual-drug HPCH system offered a promising strategy for cartilage repair by overcoming key challenges in stem cell recruitment and differentiation.

Rheumatoid arthritis (RA) is a chronic autoimmune disorder driven by dysregulated immune responses. These responses induce persistent synovial inflammation, which clinically presents as joint stiffness, pain, and swelling, ultimately leading to progressive cartilage and bone destruction [125,126]. Toll-like receptor 4 (TLR4)-mediated signaling plays a central role in RA’s immune pathogenesis [127]. Targeted delivery of receptor inhibitors through advanced systems may offer therapeutic benefits for RA. The Lee team [128] developed cyclic phage-display-derived inhibitory peptides (CP) as TLR4 antagonists, which were subsequently conjugated to hyaluronic acid (HA-CP) and incorporated into methoxy polyethylene glycol-b-poly(ε-caprolactone)-ran-poly(lactide) (PC) hydrogel to form the composite material PC + (HA-CP). Compared with the physically blended PC + (HA + CP) material, the chemically conjugated formulation demonstrated prolonged in vivo retention time and enhanced CP persistence. In preclinical arthritis models, this system demonstrated significant anti-inflammatory effects, as evidenced by the marked suppression of pro-inflammatory mediators and the enhanced restoration of the cartilage ECM. These findings establish a robust methodological framework for the targeted intra-articular delivery of therapeutic peptides, addressing critical challenges in pharmacology and expanding the therapeutic landscape for degenerative joint disorders. The scope and organization of thermosensitive hydrogels in cartilage tissues are shown in Table 3.

### 4.3. Application of Thermosensitive Hydrogels in Other Tissues

#### 4.3.1. Skin Injuries

The skin, as the largest organ of the human body, is crucial for maintaining physiological homeostasis and protecting internal organs from external insults. It serves as the primary barrier against external pathogens [139]. Furthermore, skin injuries are often accompanied by inflammatory responses, which further complicate the repair process. The highly adhesive thermosensitive hydrogel delivery system is commonly used for skin defect repair, and the administration methods are diverse (such as injection, application, spraying, or dressing) [140].

Crocin-1 (CRO-1), an active bioactive compound extracted from Crocus sativus L., exhibits potent anti-inflammatory and antioxidant properties. Lv et al. [141] prepared a CRO solution and incorporated it into hydroxybutyl chitosan (HBC) solution, followed by water bath heating to form CRO-HBC hydrogel. This material initially exhibited a burst release of CRO-1 to regulate ROS levels at the wound site, followed by sustained release to prolong the therapeutic efficacy in full-thickness burn wounds. Additionally, the combination of therapeutic agents and metal ions offers synergistic benefits for treating chronic, non-healing wounds, such as those associated with diabetes. A MC/carboxymethyl chitosan(CMC)-based thermosensitive hydrogel incorporating metal–organic framework ZIF-8 loaded with curcumin (Cur) and Zn^2+^ has been reported [142]. This hydrogel formed a protective gel barrier at temperatures ≥28 °C and exhibited controlled release of Cur in response to the wound’s acidic environment (pH 5.2). The experimental results demonstrated enhanced diabetic wound healing through a coordinated therapeutic cascade that reduced oxidative stress and modulated immune responses by switching macrophage phenotypes.

In skin wound repair, sprayable gelation provides an efficient and practical therapeutic approach. Pan et al. [143] designed a photothermal hydrogel system by integrating ILGA nano-hybrids—liposome-encapsulated imipenem modified with a gold shell and lipopolysaccharide aptamer—into a PLGA-PEG-PLGA hydrogel matrix. The composite material forms a sprayable gel that solidifies rapidly under NIR-II laser irradiation, exhibiting hemostatic, antibacterial, and anti-infective effects with promising clinical utility.

#### 4.3.2. Tumor

Malignant tumors, as a major global public health challenge, exhibit limited therapeutic efficacy with conventional combination therapies in clinical practice [144]. The core challenge lies in achieving an optimal spatiotemporal distribution of free drug molecules in vivo and precisely coordinating the co-delivery and controlled release of multiple drugs at targeted sites [145]. In recent years, thermosensitive hydrogels have been combined with nanomaterials. Through intratumoral injection, subcutaneous injection, and perfusion, the materials are implanted, effectively achieving controlled drug release and bringing new hope to tumor treatment [146].

**Figure 8:**
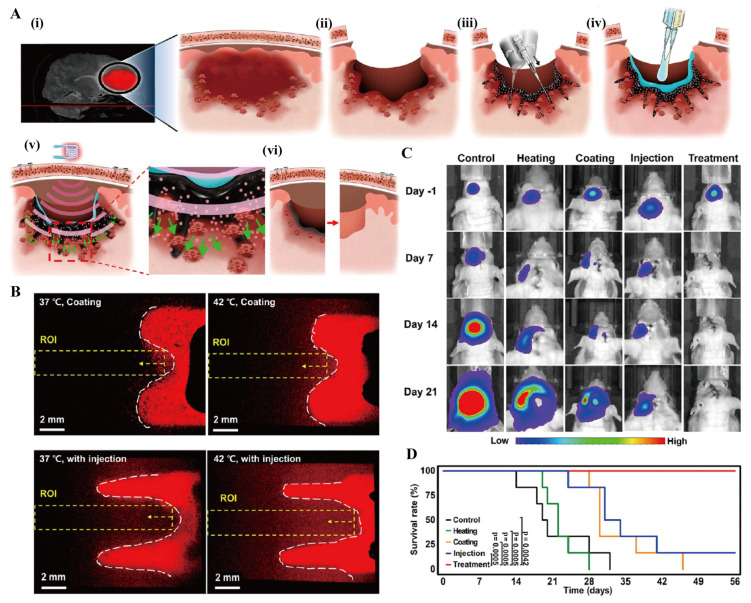
Hydrogel nanocomposite removes deeply infiltrating tumor cells. (**A**) Schematic diagram of the GBM treatment procedure using a hydrogel nanocomposite. (**i**) Detection of GBM, (**ii**) surgical removal, (**iii**) injection material, (**iv**) bioglue coating reduces drug leakage, (**v**) periodic magnetic activation under mild hyperthermia, and (**vi**) biodegradation.(Green arrows indicate the diffusion of micelles and drugs from hydrogel nanocomposite into the postoperative margin in the deep brain region.) (**B**) Fluorescence observation of the diffusion of Dox after nanocomposite coating and injection(red) onto the surface of artificial tissues(black) at different temperatures. (**C**) The results of luminescence imaging of the GBM volume after treatment (Control, injection of PBS; Heating, injection of the material without drug but with mild hyperthermia; Coating, coating of the material without mild hyperthermia; Injection, injection of the material without mild hyperthermia; and Treatment, injection of the material with mild hyperthermia. All groups underwent surgical resection of GBM). (**D**) Kaplan–Meier survival analysis for each group (by log-rank test). Reprinted with permission from [148]. Copyright 2023, American Chemical Society.

Li et al. [147] innovatively developed a thermosensitive hydrogel/nanogel composite system (Gel/REG + NG/LY). This system was formed by incorporating both transforming growth factor β inhibitor LY3200882 (LY) encapsulated in nanogels and free regorafenib (REG) into a methoxy poly(ethylene glycol)-block-poly(l-alanine) hydrogel, thereby establishing a foundation for sequential drug release. Studies demonstrated that this material preferentially releases REG to suppress tumor proliferation while upregulating ROS levels in the tumor microenvironment, subsequently triggering the responsive release of LY to block immune evasion pathways. This cascade release strategy remodels the tumor immune microenvironment by modulating macrophage polarization and T-cell infiltration, thereby significantly inhibiting both primary tumor progression and distant metastasis. Furthermore, certain tumors, such as glioblastoma (GBM), exhibit poor responsiveness to postoperative radiotherapy and chemotherapy. Kang et al. [148] used poly(lactic acid)-poly(ethylene glycol)-poly(lactic acid) hydrogel as the base and loaded doxorubicin (Dox) and T7 peptide through the nanoprecipitation method and grafting techniques. They also added water-rich ferromagnetic iron oxide nanotubes to the gel to prepare a hydrogel nanocomposite. Following administration at the tumor resection site, this system rapidly forms a deep-seated drug depot, and under mild hyperthermia, its unique magnetic field-responsive properties enable controlled deep-tissue drug penetration. This hydrogel nanocomposite has improved the survival rate of the GBM model, providing an innovative solution for removing deep-infiltrating tumor cells after surgery (Figure 8). Low-grade non-muscle-invasive bladder cancer (LG-NMIBC) is characterized by the absence of tumor cell invasion into the deep layers of the bladder wall. The current standard treatment, transurethral resection of the bladder tumor, carries risks including bladder injury, postoperative hemorrhage, and frequent recurrence. Stover et al. [149] evaluated UGN-102, a thermosensitive PLGA-PEG-PLGA hydrogel encapsulating mitomycin, administered via intravesical instillation as a non-surgical alternative. This sustained-release system enhanced local drug exposure while minimizing systemic effects. Follow-up assessments revealed no abdominal distension or flatulence, with a 65% complete response rate at three months and no observed recurrence. As an effective, low-toxicity, non-surgical first-line therapy, UGN-102 gel therapy provides a novel therapeutic option for cancer treatment.

#### 4.3.3. Tendon–Bone Interface

**Figure 9:**
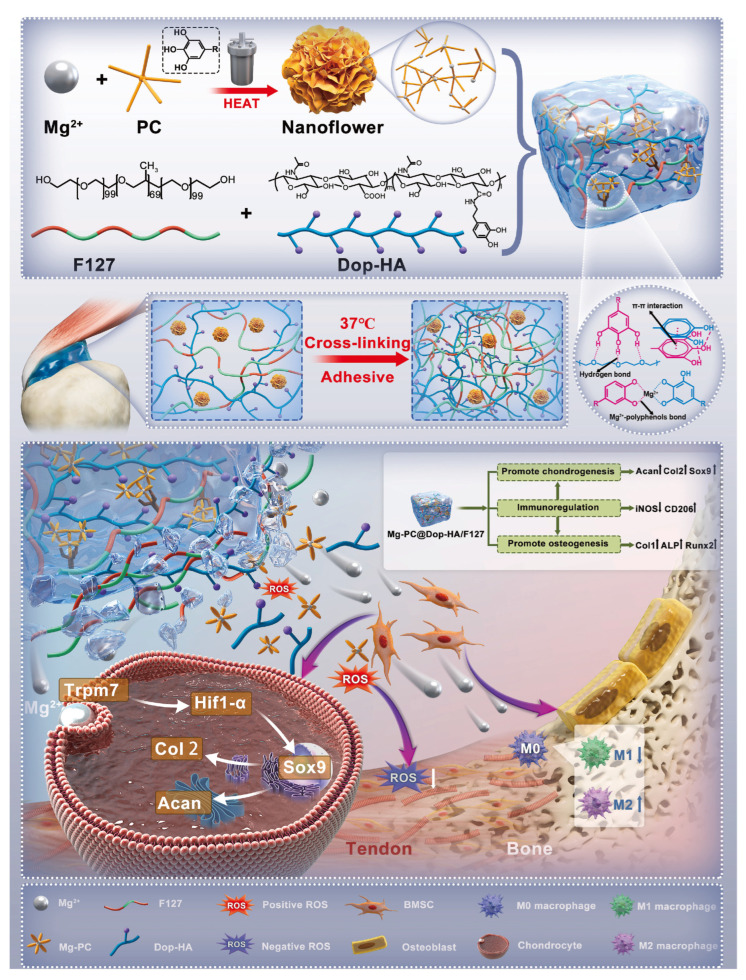
Schematic diagram of Mg^2+^ crosslinking mode and mechanism of action of Mg-PC@Dop-HA/F127 hydrogel. Reproduced with permission from Ref. [151], Elsevier.

The tendon–bone interface is a crucial junction between muscle and bone, maintaining the body’s motor function. The postoperative inflammatory environment after traditional surgery often leads to delayed remodeling of the tendon–bone interface [150]. Through metal coordination crosslinking, thermosensitive hydrogels capable of sustained metal ion release offer a promising strategy for accelerating tendon–bone interface repair. Li et al. [151] used F127 and dopamine-modified HA as the base, incorporating magnesium-procyanidin coordinated metal polyphenol nanoparticles (Mg-PC). Crosslinking Mg-PC through catechol coordination bonds within the hydrogel further enhanced the material’s mechanical strength, adhesiveness, and sustained ion release. Studies demonstrated that the release of procyanidins promotes M2 macrophage polarization. Additionally, magnesium ions facilitate cartilage matrix production through the TRPM7 channel protein. The therapeutic effect of this material was verified by injecting it into the shoulder tendon tear site of rats, providing a practical approach to accelerate the recovery of the tendon–bone interface after reconstructive surgery (Figure 9).

#### 4.3.4. Muscle

**Figure 10:**
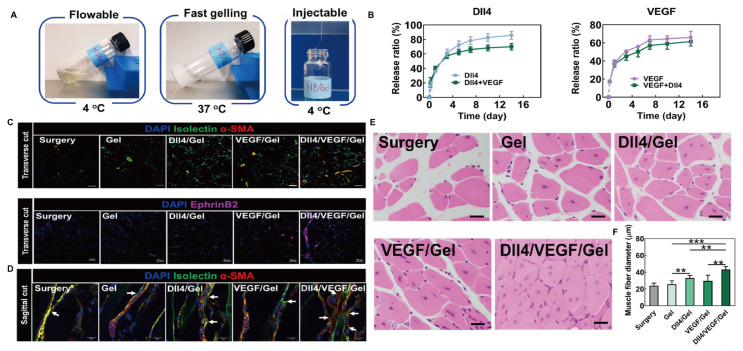
DII4/VEGF/Gel hydrogel for muscle tissue engineering. (**A**) Schematic diagram of DII4/VEGF/Gel gelation and injection. (**B**) The in vitro release rates of DII4 and VEGF. (**C**) The transverse cut of ischemic hind limb tissues was stained with isolectin, α-SMA, and EphrinB2, to observe the formation of blood vessels and small arteries 14 days after surgery (isolectin, α-SMA, and EphrinB2 are the markers of endothelial cells, vascular smooth muscle cells, and arterial endothelial cells, respectively). Scale bar = 50 μm. (**D**) The sagittal cut of ischemic hind limb tissues was stained with isolectin, α-SMA to observe the number of vessel branches 14 days after surgery (White arrows indicate the vessel branching.). Scale bar = 40 μm. (**E**) H&E staining of ischemic limb tissue samples at 14 days. Scale bar = 20 μm. (**F**) Quantitative analysis of muscle fiber diameter. ** *p* < 0.01, *** *p* < 0.001. Reprinted from Acta Biomaterialia, 143, Hong Niu(s), Delivery of VEGF and delta-like 4 to synergistically regenerate capillaries and arterioles in ischemic limbs, 15., Copyright (2022), with permission from Elsevier [154].

Peripheral artery disease is characterized by its most severe manifestation, limb ischemia, which leads to widespread skeletal muscle damage and alterations in homeostasis [152]. Bioactive substances such as GFs can promote vascular regeneration, but the efficacy of systemic administration is limited [153]. The thermosensitive hydrogel delivery system is usually administered by injection or implantation, which is more convenient and practical. Niu et al. [154] employed benzoyl peroxide as an initiator to polymerize PNIPAm, 2-hydroxyethyl methacrylate radically, and acrylated 4-(hydroxymethyl)-phenylboronic acid pinacol ester, forming an injectable thermosensitive hydrogel carrier. This carrier encapsulates VEGF and the Notch ligand Delta-like 4 (DII4). During gel degradation, the gradual release of encapsulated VEGFR2, Notch-1, and Erk 1/2 activated relevant signaling pathways, promoting robust microvascular formation in ischemic limbs. In a clinical mouse model of critical limb ischemia, this approach significantly promoted the proliferation of muscle cells, effectively increased muscle fiber diameter, and facilitated muscle tissue regeneration, demonstrating great potential for clinical application (Figure 10).

#### 4.3.5. Nerve

Spinal cord injury (SCI), a severe central nervous system pathology, induces primary neuronal death and subsequent tissue cavitation [155]. The thermosensitive hydrogel’s neural tissue-matching elastic modulus prevents nerve compression while facilitating drug delivery through injection or nasal administration. This combination of mechanical compatibility and sustained release properties offers a minimally invasive therapeutic approach for SCI.

The induction of stem cell differentiation into neurons holds great promise for treating SCI. Albashari et al. [156] engineered a thermosensitive injectable hydrogel based on F127, denoted as PF-OMSF@JK. This hydrogel achieved controlled and sustained release of H_2_S by incorporating octylsilane-functionalized mesoporous silica nanoparticles with the micromolecule H_2_S donor, JK. This biomimetic system demonstrated optimal mechanical compatibility with neural tissue and maintained excellent viability of encapsulated dental pulp stem cells (DPSCs). The continuous release of H_2_S modulates the expression of genes related to neuronal differentiation, thereby inducing the differentiation of DPSCs into neurons. Essentially, it achieves a driving effect by controlling local inflammation, which is conducive to attenuating neuroinflammation and repairing neural tissue in rat SCI models.

Similarly, Wang et al. [157] developed a hydrogel by combining GNA12/GNA13-overexpressing exosomes from engineered macrophages with CS/β-GP, creating an intranasally deliverable material. The hydrogel forms a sustained-release depot at the nasal mucosa, thereby avoiding hepatic and renal clearance and bypassing the blood brain barrier. These exosomes induce macrophage reprogramming toward anti-inflammatory M2c phenotypes. G12G13MExos@Hydrogel significantly reduced neuronal apoptosis while enhancing V2a interneuron differentiation from neural stem cells. This approach achieved effective spinal cord injury repair through a clinically feasible administration method. The scope and organization of thermosensitive hydrogels in other tissues are shown in Table 4.

## 5. Challenge and Prospects

Thermosensitive hydrogels undergo sol–gel transitions in response to temperature variations, offering a tunable and mild response mechanism. Their precise spatiotemporal control enhances the efficiency of drug delivery while facilitating targeted release at pathological sites. These properties position thermosensitive hydrogels as a versatile platform for biomedical applications. Despite the remarkable potential of thermosensitive hydrogels in tissue engineering applications, their performance varies significantly across different tissue repair scenarios. To systematically evaluate their key advantages and major challenges, this review summarizes the core characteristics of thermosensitive hydrogels in common tissue repair and treatment (Table 5). This comparative analysis highlights some common issues in thermosensitive hydrogel materials, namely, stability, biological safety, and degradation rate. For thermosensitive hydrogels, the incorporation of nanomaterials or surface coatings can enhance their structural stability [188], while expanding in vivo studies across multiple species remains crucial to validate material safety.

Currently, four-dimensional (4D) bioprinting represents a significant advancement in the development of thermosensitive hydrogels. This innovative technique builds upon conventional 3D printing by incorporating materials that respond to environmental stimuli, such as temperature, humidity, or light, thereby facilitating autonomous shape transformation and structural reconfiguration [189]. Thermoresponsive hydrogel-based bioinks serve as key components in 4D printing systems. Nakamura et al. [190] engineered a granular HA ink exhibiting temperature-dependent phase transitions, which permitted controlled sequential actuation in multimaterial constructs. The porous structure of the material undergoes reversible changes in response to temperature variations, while maintaining stable deformation capabilities over repeated heating and cooling cycles, with mechanical properties optimized to the greatest extent. Similarly, Li et al. [191] utilized shear forces during extrusion-based printing to align short carbon fibers within PNIPAm. Upon temperature changes, the hydrogel exhibited differential swelling and shrinkage rates parallel and perpendicular to the carbon fiber orientation. This anisotropic behavior generated internal stresses, allowing flexible modulation of the hydrogel’s deformation pathway. Thermosensitive hydrogels integrated with 4D printing have shown particular promise for tissue engineering applications. Wang et al. [192] developed a CS/CMC crosslinked bioink, where temperature variations triggered the scaffold’s transformation from its printed state into a structure matching corneal curvature. The method resolved uneven pore distribution in traditional hydrogels while optimizing cell loading efficiency. Temperature-induced gelation allowed in situ corneal adaptation without sutures, providing both mechanical support and tunable degradation rates for stable tissue regeneration.

We anticipate broader applications of 4D-printed thermosensitive hydrogels in tissue engineering repair, leveraging their superior shape-morphing capability and structural stability to develop more advantageous clinical treatment strategies. However, further developments necessitate collaborative efforts among researchers in tissue engineering, medicine, and additive manufacturing to optimize formulations, enhance scalability, and promote the continued evolution of thermosensitive hydrogels.

## 6. Conclusions

This review summarizes recent research progress in thermosensitive hydrogels, elucidating the gelation mechanisms of common thermosensitive hydrogel systems. Multiple optimization strategies are proposed to enhance their adhesion, mechanical strength, and multifunctionality. In the applications of bone, cartilage, and other tissue repair, we have particularly emphasized the mechanism of the thermosensitive hydrogel delivery system in substance delivery and controlled release, as well as its therapeutic effects in practical applications, highlighting its potential for development. Finally, potential development strategies are provided to address the current limitations of thermosensitive hydrogel materials. Looking ahead, the development of smarter and more responsive thermosensitive hydrogels holds great promise for advancing tissue healing and regeneration.

## Figures and Tables

**Table 1:** Comparison of different crosslinking methods of thermosensitive hydrogel.

	Physical Crosslinking	Chemical Crosslinking	Metal Coordination Crosslinking	Photothermal Synergistic Crosslinking
Crosslinking mechanism	Hydrogen bonding/electrostatic force/hydrophobic association	Covalent bond	Ion–ligand coordination	The photothermal agent absorbs near-infrared light to generate heat locally, triggering the crosslinking of the thermosensitive groups
Thermoresponsive	Reversibility	Irreversibility	Reversibility	Irreversibility/reversibility
Gelation time	Long	Short	Controllable	Controllable
Self-healing capacity	Strong	Medium	Strong	Strong
Mechanical strength	Poor	Strong	Secondary strong	Strong
Controlling release	Burst effect	Slow release	Intelligent controlled-release (pH or redox)	Precise release
Biocompatibility	Good	Medium	Consider different metal ions	Good

**Table 2:** Thermosensitive hydrogels for bone tissues.

Material Name	Highlighting the Advantages	Gelation Conditions	Application	Refs.
CSP-LB	Angiogenesis; osteoinductivity	32.7 °C	Cranial defects	[98]
sEV@CS/β-GP	Angiogenesis	37 °C	Cranial defects	[95]
Atsttrin-loaded hydrogel	Anti-inflammation	37 °C	Femoral fracture	[109]
SFD/CS/ZIF-8@QCT	Anti-inflammation; antibacterial effect; osteogenesis; stop bleeding; cell recruitment	37 °C	Periodontitis bone defects	[104]
Hydrogel + EPO + FK506	Anti-inflammation	37 °C	Periodontitis bone defects	[110]
PCc/CPO/AsA/sEVs hydrogel	Dual antibacterial effect; osteogenesis	37 °C	Periodontitis bone defects	[94]
bFGF/Dex lipo-gel	Angiogenesis; osteogenesis	31/32 °C	Cranial defects	[101]
MCP10 + GF	Angiogenesis; innervation	37 °C	Cranial defects	[111]
DCB/GP	Mechanical stability; osteogenesis	37 °C	Radius defects	[74]
CoQ10/collagen hydrogel	Anti-inflammation	25 °C	Extraction sockets of type II diabetic patients	[107]
PF127-BIO	Anti-inflammation	37 °C	Periodontitis bone defects	[103]
CGF/Gel	Osteogenesis	37 °C	Radius defects	[112]
FA-Qu-MEs@Gel	ROS scavenging ability; anti-inflammation	37 °C	Periodontitis bone defects	[113]
MSC-EC-F127DA	Osteogenesis; angiogenesis	25 °C/405 nm light source for 30 s	Extraction sockets	[92]
Hydrogel + Lentiv-GFP-PDGFBB	Osteoinductivity	37 °C	Cranial defects	[114]
DEX@CHAp/Res@CHAp/CoI I/PLEL	Anti-inflammation	37 °C	Femoral defects	[115]
T2DM/PPP-MM-S	ROS scavenging ability; osteogenesis	35 °C	Diabetic periodontal bone defects	[116]
OVX-Inf-DVG	Anti-inflammation; treatment of osteoporosis	37 °C	Femur metaphyseal fracture	[117]
GB@SIS	Anti-inflammation	37 °C	Diabetic bone defect	[108]
2Mg@PEG-PLGA gel	ROS scavenging ability; anti-inflammation	37 °C	Osteoporotic bone defects	[118]

**Table 3:** Thermosensitive hydrogels for cartilage tissues.

Material Name	Highlighting the Advantages	Gelation Conditions	Application	Refs.
^177^Lu/AMP@CG	Cartilage protection; radionuclide retention	37 °C	OA	[122]
MPA@MS@CS/SF	Mechanical stability; biocompatibility	37 °C	Articular cartilage defects	[129]
HSDKN	Stem cell recruitment; chondrogenic differentiation	37 °C	Articular cartilage defects	[124]
D-PDP@MC-Gel	Fibrocartilage hyalinization; fibrosis inhibition	37 °C	Articular cartilage defects	[130]
PF127/GlcNAc	Cartilage protection; autophagy promotion	37 °C	Articular cartilage defects	[123]
Exo@Gel	Anti-inflammation	37 °C	OA	[131]
Exo-Gel	Local retention of exosomes	37 °C	OA	[132]
p(VCL-co-HEMA)	Osteogenesis; cell sheet engineering	37 °C	Bone and cartilage defects	[133]
αApoE-loaded hydrogel	Endochondral ossification	37 °C	Bone fracture	[134]
PC + (HA-CP)	Anti-inflammation	37 °C	RA	[128]
HA/PLL-ACM/TH	Chondrogenic differentiation	37 °C	Osteochondral defects	[135]
Hyd CS/PF/BGP	Anti-inflammation	37 °C	OA	[136]
CS1-PF25-TPP	High drug retention rate	37 °C	OA	[137]
DMM + Gel@EX^modAtf5^	Activate chondrocyte autophagy; maintain mitochondrial function	37 °C	OA	[138]

**Table 4:** Thermosensitive hydrogels for other tissues.

Material Name	Highlighting the Advantages	Gelation Conditions	Application	Refs.
CS/GP/HPC–GO–PPR	Antibacterial effect	37 °C	Infected skin wounds	[158]
BP@Gel	Antibacterial effect; angiogenesis; collagen deposition	37 °C	Infected skin wounds	[159]
CS + ASCs-Exos	Sustained exosome release; synergistic immunomodulation	37 °C	Burned skin wounds	[160]
DHM-OTH	Oxygen-producing	37 °C	Diabetic skin wounds	[161]
LL37@ZPF-2 Gel	Antibacterial effect; high encapsulation	28 °C	Infected skin wounds	[162]
PF-OMSF@JK/DPSCs	Regulating and inducing stem cell differentiation	37 °C	Spinal cord injury	[156]
PFLD hydrogel	Antibacterial effect; antioxidant properties	33 °C	Infected skin wounds	[163]
Gel + TNF-R1 NV	Anti-inflammation	37 °C	Burned skin wounds	[164]
12%Hypo(rhNGF)	Nerve regeneration	37.3 °C	Neurogenic keratitis	[165]
CIC Gel	Anti-inflammation	37 °C	Muscle defect in sepsis	[166]
PH-127/PH	Skin cell proliferation; antibacterial effect	37 °C	Infected skin wounds	[167]
PF-127 + HUCMSCs + SAP	Antibacterial action; angiogenesis; cell proliferation	37 °C	Infected skin wounds	[168]
HAP/PDA/PF-127	Nerve neovascularization	37 °C	Sciatic nerve injury	[169]
Ni_4_Cu_2_/F127	Enzyme-like activity	37 °C	Skin wounds	[170]
DPSCs-PF127	Angiogenesis	37 °C	Fallopian tube mucosa defects	[171]
Lfcin-Nio/HG	Antibacterial effect;	33–37 °C	Infected skin wounds	[172]
H-hUCESC-CM	anti-inflammation	37 °C	Inflammatory bowel disease	[173]
CME-AgNPs-F127/F68	Antibacterial effect	37 °C	Infected skin wounds	[174]
PTX-NCS-gel	Antineoplastic	37 °C	Breast cancer	[175]
Mg-PC@Dop-HA/F127	Anti-inflammation; ROS scavenging ability	37 °C	Tendon–bone interface	[151]
DII4/VEGF/Gel	Cell proliferation; angiogenesis	37 °C	Limb ischemia	[154]
PNI/RA-Amps3/E	Angiogenesis; fibroblast proliferation	32 °C	Skin wounds	[176]
p(NIPAM-co-HEMIN)/ALG-EDA/AgNPs	Antibacterial effect; antioxidant dual-function	37 °C	Diabetic skin wounds	[177]
TQ@PEG-PAF-Cur	Bacterium-targeted ROS generation	30 °C	Infected skin wounds	[178]
CRO-HBC	Anti-inflammation; ROS scavenging ability	37 °C	Burned skin wounds	[141]
HBCA3	Antibacterial effect	48 °C	Infected skin wounds	[179]
Implant + Hydrogel + Cisplatin	Antineoplastic	37 °C	Osteosarcoma	[180]
Hydrogel nanocomposite	Removal of deep tumor cells	37 °C	Postoperative treatment of glioblastoma	[148]
MCC@ZIF-8@Cur	Anti-inflammation; ROS scavenging ability	28 °C	Diabetic skin wounds	[142]
UCMSC-bFGF-ECM-HP	Regulation of mitochondrial function	37 °C	SCI	[181]
QTF@PNAGA hydrogel	Anti-tumor properties	47 °C	Skin cancer	[182]
Gel + NEP1-40	Protect neurons; promote axon growth	37 °C	Brachial plexus nerve root avulsion injury	[183]
LPS/PPP	Anti-tumor properties	37 °C	Colorectal cancer	[184]
UGN-102	Anti-tumor properties	37 °C	LG-NMIBC	[149]
G12G13MExos@Hydrogel	Anti-inflammation; neural stem cell differentiation	34 °C	SCI	[157]
5FU-Alg-Np-HG	High drug retention rate	34 °C	Skin cancer	[185]
ASGP/SA/PNIPAM	Cell proliferation	37 °C	Diabetic skin wounds	[186]
ILGA@Gel	Hemostasis; anti-inflammation; antibacterial effect; avoid reinfection	37 °C	Skin wounds	[143]
PPP + CNTF	Anti-inflammation; antioxidant; neuroprotection	37.5 °C	Optic nerve	[187]

**Table 5:** The advantages and challenges of thermosensitive hydrogels for tissue repair.

Tissue Type	Key Advantages	Major Challenges
Bone	Adaptability of irregular bone defect shapes and treatment; slow release of GFs and anti-inflammatory drugs; no need for a second operation	Insufficient mechanical strength for large-segment bone defect repair; the degradation and regeneration rates are Difficult to match; insufficient long-term stability
Cartilage	Minimally invasive injection; sustained intra-articular drug release; enhance targeting ability; no need for a second operation	Insufficient long-term stability; low integration efficiency with native tissue
Skin	Simple operation; multiple response; combined antimicrobial/anti-inflammatory action	Unstable tissue adhesion; vulnerability to infection
Tumor	Minimally invasive; localized high-dose delivery; enhance targeting ability; photothermal combination; magnetic-thermal combination	Insufficient long-term stability; carrier biocompatibility issues; insufficient clinical validation
Tendon–bone interface	Minimally invasive; enhance targeting ability; no need for a second operation	Dynamic stress-induced interface failure; insufficient long-term stability
Muscle	Minimally invasive; no need for a second operation	Material displacement during contraction; limited efficacy in large-volume defect repair; the degradation and regeneration rates are difficult to match; insufficient long-term stability
Nerve	Mechanical compatibility; no need for a second operation	Blood–nerve barrier restrictions; insufficient long-term stability
